# Strengthening primary health care service competency: a scoping review of challenges, influencing factors, and enhancement strategies

**DOI:** 10.3389/fpubh.2025.1732011

**Published:** 2026-01-09

**Authors:** Sen Yang, Chaohui Bai, Yajun Zhao, Jiaojiao Wang, Hua Jin, Ben Harris-Roxas, Yalin Liu, Fei Shen, Huaxin Zhao, Le Ma, Leiyu Shi, Dehua Yu

**Affiliations:** 1 Department of General Practice, Yangpu Hospital, School of Medicine, Tongji University, Shanghai, China; 2 Daqiao Community Healthcare Centre, Shanghai, China; 3 Department of Health Management Centre, Zhongshan Hospital, Fudan University, Shanghai, China; 4 Shanghai General Practice and Community Health Development Research Center, Shanghai, China; 5 School of Population Health, University of New South Wales Faculty of Medicine, Sydney, NSW, Australia; 6 Department of General Practice, Community Health Service Center of Jiading Town in Jiading District, Shanghai, China; 7 Changbai Community Health Service Center, Shanghai, China; 8 Department of Oncology, Shanghai Tenth People's Hospital, Tongji University School of Medicine, Shanghai, China; 9 Johns Hopkins Primary Care Policy Center, Baltimore, MD, United States

**Keywords:** capability enhancement, challenges and strategies, health systems strengthening, healthcare policy, influencing factors, primary health care, scoping review, universal health coverage

## Abstract

**Objective:**

This scoping review aims to evaluate the factors influencing the service capacity of primary health care (PHC) institutions and to identify strategies for their development.

**Methods:**

We conducted a scoping review based on Arksey and O’Malley’s framework and systematically searched PubMed, Web of Science, and SCOPUS for studies published from January 2014 to December 2024. Eligible research addressing PHC capacity-building interventions across diverse health systems was synthesized thematically.

**Results:**

This scoping review synthesized evidence from 76 publications (2014–2024) addressing PHC competency enhancement, comprising original research articles (63.2%, *n* = 48) and systematic/scoping reviews (36.8%, *n* = 28). Key challenges identified included workforce shortages, inequitable resource allocation, fragmented policy frameworks, limited integration of health information technologies, and persistent inequities in service delivery. Strategies to strengthen PHC capacity centered on workforce development, technology-enabled innovation, equitable service models, and interdisciplinary collaboration.

**Conclusion:**

Strengthening PHC capacity requires integrated approaches, including localized workforce training, technological adoption, optimized equity in service provision, and enhanced interdisciplinary cooperation. These approaches provide context-sensitive and equity-oriented pathways for building sustainable PHC systems—particularly in resource-constrained settings—supporting progress toward Universal Health Coverage and reducing global health disparities.

**Systematic review registration:**

Registration DOI: 10.17605/OSF.IO/MDR73.

## Introduction

The efficacy of primary health care (PHC) institutions is foundational to building resilient and equitable health systems globally (1). Despite significant advancements in PHC service capacity, particularly in underserved and rural areas (2, 3), a critical gap remains in the effective implementation of capacity-building strategies tailored specifically to PHC institutions. This gap is exacerbated globally, as health systems face extensive demands and resource constraints across regions (4). In this context, systematically analyzing the multidimensional challenges and strengthening paths of PHC service capacity is of strategic significance for realizing the goal of universal health coverage (UHC).

The efficacy of PHC institutions is foundational to building resilient and equitable health systems globally, as underscored by the World Health Organization (WHO) and key frameworks such as the Astana Declaration (5). In this context, systematically analyzing the multidimensional challenges and strengthening paths of PHC service capacity is of strategic significance for realizing the goal of UHC. This capacity covers core functions such as disease prevention, early diagnosis, and chronic disease management, and is the first line of defense in responding to the complex needs of population health. However, most existing studies have focused on macro health system reform or specialty healthcare innovation (6, 7), ignoring the specificity of capacity building in primary healthcare organizations. This oversight is significant, given that PHC institutions frequently serve as the first point of contact for patients and play a pivotal role in early detection, disease prevention, and management. Unlike prior meta-analyses, which have generally addressed broader health system challenges, this review uniquely focuses on the multidimensional factors affecting the capacity of PHC institutions and systematically synthesizes strategies for capacity building across diverse contexts (8, 9). More importantly, there is a lack of synthesizing studies that systematically sort out the challenges and strategies for capacity building of PHC services in different geographic regions, socio-economic contexts and health systems (1, 3, 4).

Given these global challenges and the critical role of PHC in advancing UHC, this scoping review aims to critically evaluate the current state of capacity-building strategies for essential health services in PHC institutions. Specifically, it seeks to address the following research questions:

1. What are the major challenges and barriers to PHC service capacity?
2. What are the key factors affecting PHC service capacity?
3. What strategies have been proposed in existing studies to enhance PHC service capacity?

## Methods

Given the heterogeneous nature of evidence and the exploratory focus on mapping challenges and strategies for strengthening PHC service competency, a scoping review methodology was selected. This approach aligns with the framework proposed by Arksey and O’Malley (10), which is particularly suited to synthesizing diverse evidence types, identifying knowledge gaps, and clarifying conceptual boundaries in under-researched areas. The study protocol was prospectively registered with the Open Science Framework (OSF) on June 22, 2024 to mitigate bias and enhance reproducibility (Registration DOI: 10.17605/OSF.IO/MDR73) (11).

### Search strategy

The search strategy was designed following the Preferred Reporting Items for Systematic Reviews and Meta-Analyses Extension for Scoping Reviews (PRISMA-ScR) guideline (12) to ensure comprehensiveness and reproducibility (Appendix 1). Three major databases—PubMed, Web of Science (WOS), and Scopus—were systematically searched from January 1, 2014, and December 31, 2024. Key concepts were operationalized using a combination of controlled vocabulary (e.g., MeSH terms) and free-text keywords, with boolean operators (AND/OR) and truncation symbols (*) to capture variations, with the completed checklist provided in Appendix 2.

### Inclusion criteria and exclusion criteria

Inclusion criteria encompassed:(1) studies addressing PHC services in any geographic or socioeconomic context; (2) explicit discussion of challenges, influencing factors, or strategies for strengthening PHC competency; and (3) peer-reviewed empirical studies (quantitative, qualitative, or mixed-methods) and systematic or scoping reviews.

Exclusion criteria were: (1) studies focused on secondary/tertiary care or single-disease programs without PHC integration; (2) Gray literature (e.g., policy briefs, conference abstracts, and unpublished reports) was excluded to ensure all included evidence met peer-review standards and could be reliably appraised for methodological rigor and traceability. While recognizing that gray literature may contain relevant policy insight, this choice prioritized consistency and verifiability across global contexts; (3) inaccessible full texts despite reasonable retrieval efforts; (4) studies conducted in humanitarian crisis zones due to contextual uniqueness. See Table 1 for details.

**Table 1:** Inclusion and exclusion criteria applied in the scoping review.

Domain	Inclusion criteria	Exclusion criteria
Population/Context	Primary health care (PHC) services in any geographic or socioeconomic setting.	Studies focused on secondary/tertiary care, specialized clinics, or single-disease programs without PHC integration.
Concept	Explicitly addresses ≥1 of:	Non-analytical work (e.g., commentaries, editorials).
	PHC challenges/barriers	Animal/laboratory studies.
	Influencing factors	
	Enhancement strategies	
Study types	Peer-reviewed original research (quantitative, qualitative, mixed-methods).	Purely descriptive reports without critical analysis.
	Reviews (systematic, scoping).	
Timeframe	Published between January 2014 and December 2024.	Studies outside the 2014–2024 timeframe.
Language	English-language publications.	Non-English publications.
Data accessibility	Full text available.	Full text inaccessible despite reasonable efforts.
Geographic focus	All regions.	Studies conducted in humanitarian crisis zones (e.g., active conflict areas).

This table outlines the inclusion and exclusion criteria used to guide study selection, organized by population/context, conceptual focus, study design, timeframe, language, data accessibility, and geography. These criteria ensured a systematic and reproducible selection process.

### Data charting and validation

A structured data charting form was developed based on Arksey and O’Malley’s methodological guidance and refined through pilot testing on five studies. Data fields included study characteristics, PHC context, methodological approach, reported challenges, influencing factors, and capacity-building strategies. Two reviewers independently populated the data chart, followed by a cross-checking process. A third reviewer conducted random verification of 20% of entries. Iterative consensus discussions were held to resolve discrepancies and refine thematic categories. This multi-stage validation enhanced reliability, minimized coder bias, and ensured replicability.

## Selection process

### Title and abstract screening

Three reviewers (SY, CHB, and YJZ) applied the eligibility criteria to screen titles and abstracts for potential inclusion, ensuring that duplicate documents were identified and removed.

### Full-text screening

Two reviewers (SY and CHB) independently applied the eligibility criteria to screen the full texts for eligibility. Any disagreements between the reviewers were resolved through discussion or, if necessary, with the assistance of a third reviewer (JJW).

### Data extraction

Two reviewers (YS and CHB) independently performed data extraction, with a third reviewer (YJZ) conducting blinded quality control assessments on randomly selected samples to ensure consistency and accuracy. No significant discrepancies were identified during this process. When a relevant article cited another source, the original work was retrieved and assessed for potential inclusion, and necessary data were extracted to ensure comprehensive coverage. Care was taken to avoid duplicating data from the same study. The extraction form captured key details, including study characteristics (e.g., author, year, title, country, literature type, and methods), research themes, primary findings, challenges to strengthening PHC capabilities, factors affecting capacity-building, and strategies for enhancement.

### Data synthesis and analysis

A narrative synthesis was conducted, wherein each selected guidance resource was reviewed, and relevant data were extracted under predefined headings. The extracted data were then analyzed using content analysis and organized by frequency. The frequency of responses for each of the background and data categories from all included resources was recorded, synthesized, and presented through data visualizations. For outcomes, guidance resources often covered multiple themes, and non-mutual categorization was used to accommodate these overlapping groupings.

### Patient and public involvement

Given that this review focuses on previously completed studies, there was no involvement of patients or the public.

## Results

**Figure 1:**
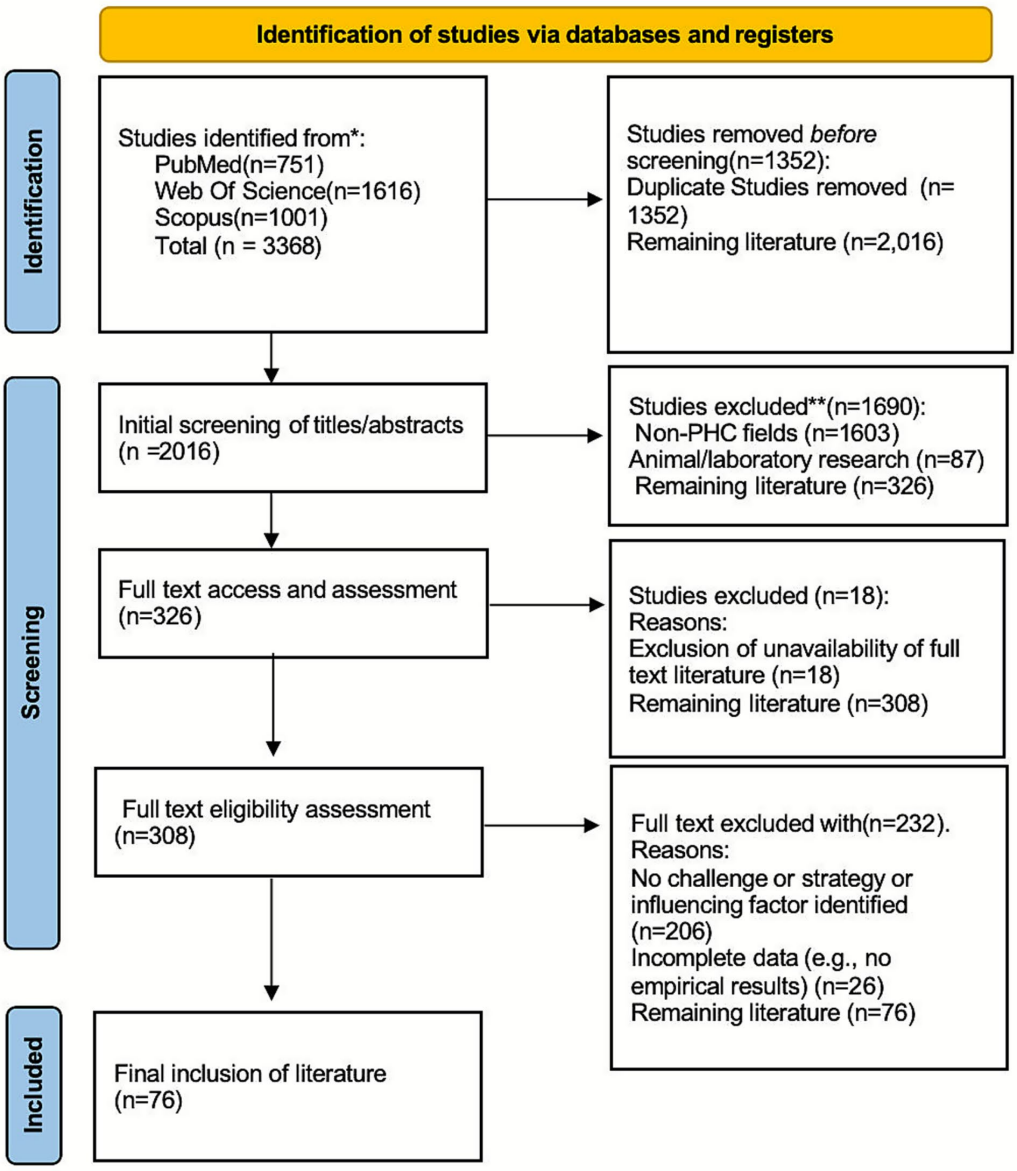
PRISMA 2020 flow diagram. The PRISMA-ScR flow diagram illustrates each stage of the study selection process in detail, including database-specific duplicates, screening outcomes, and explicit reasons for full-text exclusion. *Consider, if feasible to do so, reporting the number of records identified from each database or register searched (rather than the total number across all databases/registers). **If automation tools were used, indicate how many records were excluded by a human and how many were excluded by automation tools.

Of the 2016 titles and abstracts that were screened, 326 were included for full text screening, with 76 studies ultimately meeting the inclusion criteria. The selection workflow, including reasons for exclusion, is detailed in the PRISMA flow diagram (Figure 1). This scoping review analyzed 76 publications (2014–2024) on PHC competency enhancement, comprising original research articles (63.2%, *n* = 48) and reviews (36.8%, *n* = 28), including systematic/scoping reviews. Methodologically, studies employed qualitative (47.4%, *n* = 36), mixed-methods (27.6%, *n* = 21), and quantitative designs (25.0%, *n* = 19). Geographically, research was predominantly conducted in High-Income Countries (HICs): the United States (21.1%, *n* = 16), Canada (10.5%, *n* = 8), and Australia (9.2%, *n* = 7), with notable contributions from China (7.9%, *n* = 6). More details on the studies included in this review, such as their scope, methodology, and key findings, can be found in Table 2.

**Table 2:** Characteristics of the included literatures.

Author (s)	Year published	Title	Country	Type of literature	Research methods	Maior challenges and barriers/core influencing factors/strategies for improvement
Kuhlmann et al. (14)	2018	Primary care workforce development in Europe: an overview of health system responses and stakeholder views	Europe	Review	Qualitative study	Challenges: workforce shortages, geographical maldistribution, high outward-migration rates, lack of coordination between education and labor markets.
Spooner et al. (15)	2020	Regional variation in practitioner employment in general practices in England: a comparative analysis	United Kingdom	Original Research	Quantitative study	Challenges: resource limitations, geographical maldistribution, high outward-migration rates, lack of coordination between education and labor markets.
Maskrey et al. (16)	2018	Releasing GP capacity with pharmacy prescribing support and new ways of working: a prospective observational cohort study	United Kingdom	Original research	Quantitative study	Challenges: workforce crisis, high workload, funding constraints, recruitment issues in rural areas.
Li et al. (17)	2020	Quality of primary health care in China: challenges and recommendations	China	Review	Qualitative study	Challenges: infrastructure imbalance, declining medical insurance funds, lack of operational mechanism vitality, low salary levels, imperfect incentive systems.
Esu et al. (18)	2021	Interventions for improving attraction and retention of health workers in rural and underserved areas: a systematic review of systematic reviews	Australia	Systematic review	Mixed methods study	Challenges: health workforce shortage, maldistribution, low staff motivation, emigration of health workers.
McFarlane et al. (19)	2018	How primary health care staff working in rural and remote areas access skill development and expertise to support health promotion practice	Australia	Original research	Qualitative study	Challenges: resource limitations, geographical maldistribution, lack of continuous professional development opportunities.
Wen et al. (20)	2022	Increasing geriatric care capability in Hawai’i’s healthcare systems through the pacific islands geriatrics workforce enhancement program (GWEP) at the University of Hawai’i	United States	Original research	Mixed methods study	Challenges: COVID restrictions, staff turnover, inability to expand programs.
Tangcharoensathien et al. (22)	2018	Health systems development in Thailand: a solid platform for successful implementation of universal health coverage	Thailand	Review	Qualitative study	Challenges: funding shortage, personnel shortage, data system imperfection.
Sirkin et al. (23)	2023	Primary care’s challenges and responses in the face of the COVID-19 pandemic: insights from AHRQ’s learning community	United States	Original research	Quantitative study	Challenges: revenue loss, resource constraints, telehealth implementation difficulties, burnout, limited engagement with public health.
Bierman et al. (24)	2022	Realizing the dream: the future of primary care research	United States	Original research	Qualitative study	Challenges: clinician burnout, lack of trust in health system, evidence gaps, health inequities.
Toukhsati et al. (25)	2024	Burnout and retention of general practice supervisors: prevalence, risk factors and self-care	Australia	cross-sectional study	Quantitative study	Challenges: resource shortage, personnel shortage, heavy workload, data system imperfection.
Mullan et al. (26)	2023	Barriers and enablers to structured care delivery in Australian rural primary care	Australia	Review	Qualitative study	Challenges: workforce shortages, limited services and capacity, cultural issues, lack of resources, poor coordination, unclear roles.
Mathews et al. (27)	2024	System-based interventions to address physician burnout: a qualitative study of Canadian family physicians’ experiences during the COVID-19 Pandemic	Canada	Original research	Qualitative study	Challenges: inability to provide appropriate care, workload pressure, financial uncertainty.
Stenberg et al. (28)	2019	Guide posts for investment in primary health care and projected resource needs in 67 low-income and middle-income countries: a modelling study	67 low-income and middle-income countries (LMICs)	Original research	Quantitative study	Challenges: insufficient models for cross-sectoral investments, limited baseline data
Bolongaita et al. (29)	2023	Financial hardship associated with catastrophic out-of-pocket spending tied to primary care services in low- and lower-middle-income countries: findings from a modeling study	Low- and lower-middle-income countries	Original research	Quantitative study	Challenges: limited social protection, weak insurance, lack of data, missing inputs, and proxy metrics issues.
Weber et al. (30)	2020	Rwandan primary healthcare providers’ perception of their capability in the diagnostic practice	Rwandan	Original research	Qualitative study	Challenges: skill-mix imbalance, insufficient training, resource constraints (time, rooms, lab tests), rare and short supervision.
Endalamaw et al. (31)	2023	Successes and challenges towards improving the quality of primary health care services: a scoping review	Australia	Review	Qualitative study	Challenges: health worker shortage, geographical disparity, poor quality mental health care, lack of guidelines, inadequate information to clients.
Zhu et al. (32)	2019	What does the Chinese public care about with regard to primary care physicians: trustworthiness or competence?	China	Original research	Mixed methods study	Challenges: low public trust, insufficient physician competence, lack of medical equipment, weak popularity, insufficient communication between patients and physicians, and slow progress of primary care reform.
Etz et al. (33)	2023	Telemedicine in primary care: lessons learned about implementing health care innovations during the COVID-19 pandemic	United States	Original research	Quantitative study	Challenges: inadequate payment, lack of resources, tech issues, policy lags.
Du et al. (8)	2022	Factors influencing adoption and use of telemedicine services in rural areas of china: mixed methods study	China	Original research	Mixed Methods Study	Challenges: limited resources, technical issues, lack of training. Influencing factors: infrastructure, policy support, patient acceptance, physician willingness. Strategies: strengthen infrastructure, enhance training, improve policies, increase awareness.
Fiscella et al. (34)	2018	The complexity, diversity, and science of primary care teams	United States	Review	Qualitative study	Challenges: visit-based payment systems, limited time, unclear leadership, restrictive regulations, lack of shared mental models
Agustina et al. (35)	2019	Universal health coverage in Indonesia: concept, progress, and challenges	Indonesia	Review	Qualitative study	Challenges: Infrastructure gaps, rural–urban disparity, insufficient medical personnel, limited funding, high prevalence of non-communicable diseases, maternal mortality, childhood stunting, tuberculosis.
Khatri et al. (36)	2024	Enablers and barriers of community health programs for improved equity and universal coverage of primary health care services: a scoping review. BMC Prim Care	Australia	Scoping Review	Qualitative study	Challenges: inadequate funding, limited private sector engagement, poor service quality, low focus on NCDs, CHWs’ skill and funding shortages for NCDs.
Shahidi et al. (93)	2019	The impact of social assistance programs on population health: a systematic review of research in high-income countries.	Canada	Systematic review	Qualitative study	/
Ford et al. (37)	2019	Improving primary care access in context and theory (I-ACT trial): A theory-informed randomised cluster feasibility trial using a realist perspective	United Kingdom	Original research	Mixed methods study	Challenges: resource allocation, funding, policy support, community participation.
Ahmat et al. (38)	2022	The health workforce status in the WHO african region: findings of a cross-sectional study	Congo	Original research	Quantitative study	Challenges: human resource shortage, uneven distribution, heavy workload, insufficient training, lack of infrastructure.
Agyei et al. (39)	2024	Navigating the complex terrain of healthcare systems in Sub-Saharan Africa: challenges and opportunities for progress	Sub-Saharan Africa	Systematic review	Quantitative study	Challenges: inadequate health infrastructure, shortage of trained healthcare professionals, equity issues in access, insufficient financing and high OOP.
Ntshiqa et al. (40)	2023	Knowledge, attitudes, practices, and acceptability of medical male circumcision among males in traditionally circumcising rural communities of Alfred Nzo District, Eastern Cape, South Africa	South Africa	Original research	Quantitative study	Challenges: low MMC uptake in traditional communities, cultural resistance, potential complications of TMC.
Mengistu et al. (41)	2023	Successes and challenges of primary health care in Australia: a scoping review and comparative analysis	Australia	Review	Qualitative study	Challenges: socioeconomic disadvantage, geographic barriers, staff turnover, low person-centered care adoption, poor sectoral collaboration.
Lai et al. (42)	2024	Challenges and strategies of developing internet hospital: combining qualitative interview and documentary research	China	Original research	Mixed methods study	/
Borges do Nascimento et al. (43)	2023	Barriers and facilitators to utilizing digital health technologies by healthcare professionals. NPJ Digit	All over the world	Review	Mixed methods study	Challenges: infrastructure and technical issues, psychological barriers, workload concerns, lack of training.
Koonin et al. (44)	2020	Trends in the use of telehealth during the emergence of the COVID-19 pandemic—United States, January–March 2020	United States	Cross-sectional study	Quantitative study	Challenges: Limited access to the Internet/devices, lack of tech familiarity, inappropriateness for some patients.
Okobi et al. (45)	2023	Examining disparities in ownership and use of digital health technology between rural and urban adults in the US: an analysis of the 2019 health information national trends survey	United States	Cross-sectional analysis	Quantitative study	Challenges: lack of primary & specialist services, geographic barriers, long travel/wait times, provider shortage.
Almalawi et al. (46)	2023	Managing security of healthcare data for a modern healthcare system	Saudi Arabia	Original research	Quantitative study	Challenges: data storage and secure transfer, high cost and lengthy process, data security and privacy protection, complexity of data management.
Terry et al. (47)	2022	Is primary health care ready for artificial intelligence? What do primary health care stakeholders say?	Canada	Cross-sectional study	Qualitative study	Challenges: diagnostic accuracy, AI errors, increased workload, evidence base, algorithm bias.
Odendaal et al. (48)	2020	Health workers’ perceptions and experiences of using mHealth technologies to deliver primary healthcare services: a qualitative evidence synthesis	low and middle-income countries	Original Research	Qualitative study	Challenges: poor network and electricity access, extra workload, device damage, data entry errors.
Gray et al. (94)	2017	Building competency and capacity for promotion of effective physical activity in diabetes care in Canada	Canada	Original research	Quantitative study	Impact factors: providers’ knowledge, confidence, time availability, patients’ health status and comorbidities.
Koly et al. (50)	2021	Educational and training interventions aimed at healthcare workers in the detection and management of people with mental health conditions in South and South-East Asia: a systematic review	South and South-East Asia	Systematic review	Mixed methods study	Influencing factors: digital tech (enabling), stakeholder involvement, cost and time (enabling)
Herzog et al. (51)	2015	We can do only what we have the means for’ general practitioners’ views of primary care for older people with complex health problems	Germany	Original Research	Qualitative study	Influencing factors: remuneration modalities, geriatric qualification level, attitudes, lack of understanding of other professionals’ services.
Zhao et al. (52)	2019	Perceptions, behaviors, barriers and needs of evidence-based medicine in primary care in Beijing: a qualitative study	China	Original Research	Qualitative study	Influencing factors: patient attitude, workload, resource availability, training level, guideline quality.
Petersen et al. (55)	2023	Reducing acute hospitalizations at high-performing CPC + primary care practice sites: strategies, activities, and facilitators	United States	Original research	Mixed methods study	Influencing factors: practice transformation experience, data use, team-based approach, innovation interest.
Lim et al. (56)	2021	Implementation of a multi-level community-clinical linkage intervention to improve glycemic control among south Asian patients with uncontrolled diabetes: study protocol of the DREAM initiative	South Asians	Original research	Qualitative study	Influencing factors: cultural and linguistic barriers, patient health literacy, community resource availability, provider-patient communication.
Spehar et al. (57)	2017	General practitioners’ views on leadership roles and challenges in primary health care: a qualitative study	Norway	Original research	Qualitative study	Influencing factors: limited training opportunities, clinical workload, lack of formal leadership structure.
Herbert et al. (58)	2015	Perspectives in primary care: values-driven leadership is essential in health care	Canada	Review	Qualitative study	Influencing factors: community resource availability, patient health literacy, provider-patient communication.
Nzinga et al. (53)	2021	An innovative leadership development initiative to support building everyday resilience in health systems	Kenya	Original research	Mixed methods study	Influencing factors: unclear hierarchies, political interference, mistrust, and bureaucratic inertia.
Carr et al. (54)	2024	Strengthening healthcare providers’ leadership capabilities, interprofessional collaboration, and systems thinking: a conceptualization of the clinical scholars program impact	United States	Original research	Mixed methods study	Influencing factors: organizational inefficiencies, lack of collaborative culture, poor talent identification, resource constraints.
Mayston et al. (59)	2020	Measuring what matters—information systems for management of chronic disease in primary healthcare settings in low and middle-income countries: challenges and opportunities	low and middle-income countries	Original research	Qualitative study	Influencing factors: poor IT infrastructure, weak interoperability, lack of prioritization, insufficient decentralization, policy disruptions.
Yan et al. (60)	2021	Effectiveness of a primary care-based integrated mobile health intervention for stroke management in rural China (SINEMA): a cluster-randomized controlled trial	China	Original Research	Quantitative study	Influencing factors: poor data quality, lack of specialized knowledge and infrastructure, low prioritization of chronic disease by governments.
Saif-Ur-Rahman et al. (61)	2023	Artificial intelligence and digital health in improving primary health care service delivery in LMICs: a systematic review	in low and middle-income countries	Review	Qualitative study	Influencing factors: socioeconomic contexts, health system development.
Xu et al. (62)	2021	Chatbot for health care and oncology applications using artificial intelligence and machine learning: Systematic Review	Canada	Systematic review	Qualitative study	Influencing factors: hesitancy, algorithm bias, data quality.
Amoakoh et al. (63)	2019	Using mobile health to support clinical decision-making to improve maternal and neonatal health outcomes in Ghana: insights of frontline health worker information needs	Ghana	Original research	Qualitative study	Influencing factors: cost, distance, attitude of nurses, lack of facilities.
Clarfield et al. (64)	2017	Health and health care in Israel: an introduction	Israel	Review	Qualitative study	Influencing factors: budget reductions, uneven distribution of healthcare workers, population aging, increased prevalence of chronic diseases.
Henderson DAG, et al. (65)	2023	Understanding primary care transformation and implications for ageing populations and health inequalities: a systematic scoping review of new models of primary health care in OECD countries and China	OECD countries and China	Original research	Mixed methods study	Influencing factors: leadership, resources, culture, targets.
Aoki et al. (66)	2020	Comparison of primary care experience in hospital-based practices and community-based office practices in japan	Japan	Original research	Quantitative study	Influencing factors: practice location and ownership. Work environment and staff size.
Hill et al. (67)	2020	The effectiveness of continuous quality improvement for developing professional practice and improving health care outcomes: a systematic review	United Kingdom	Original research	Quantitative study	Influencing factors: adaptation variation, adoption as standard practice, incomplete reporting, poor evidence quality.
Tan et al. (68)	2021	Singapore’s health-care system: key features, challenges, and shifts	Singapore	Review	Mixed methods study	Influencing factors: Rising non-communicable diseases, population ageing, financial incentives.
Scott-Richardson et al. (69)	2022	Policy facilitators versus structural barriers: integrative therapy telehealth changes in the united states during the COVID-19 pandemic	United States	Original research	Quantitative study	Influencing factors: lack of broadband, social vulnerability, insurance coverage.
Carruthers et al. (70)	2023	Interventions to improve access to primary care for inclusion health groups in England: a scoping review	United Kingdom	Original research	Qualitative study	Strategies: training, education, resources, targeting GP registration, involving community organizations.
Mydin FHM, et al. (71)	2021	The effectiveness of educational intervention in improving primary health-care service providers’ knowledge, identification, and management of elder abuse and neglect: a systematic review	Malaysia	Systematic review	Mixed methods study	Strategies: training programs, continuous education, multicomponent interventions.
Seki et al. (72)	2022	Use of a 2-year continuing professional development programme to change Japanese physicians’ attitudes to learning primary care: a qualitative study	Japanese	Original Research	Qualitative study	Strategies: PBL-based CPD program, forming learning community, online program hosting.
Coales et al. (73)	2023	Perspectives of health workers engaging in task shifting to deliver health care in low-and-middle-income countries: a qualitative evidence synthesis.	United Kingdom	Review	Qualitative study	Strategies: task shifting, proper training, supervision, securing resources, aligning with values, improving workplace culture.
Wensing et al. (74)	2019	Knowledge translation in health: how implementation science could contribute more	Germany	Original research	Qualitative study	Strategies: strengthen leadership, promote knowledge co-production, establish partnerships and networks, ensure organizational readiness, contextualize evidence.
Baldoni et al. (75)	2019	Telepharmacy services: present status and future perspectives: a review	Italy	Review	Qualitative study	Strategies: telepharmacy, standardization, cooperation among sectors.
Samuel et al. (76)	2021	Effect of continuing professional development on health professionals’ performance and patient outcomes: a scoping review of knowledge syntheses	United States	Review	Mixed methods study	Strategies: adopt a multi-component approach use eLearning consider costs in design.
Alami et al. (77)	2020	Artificial intelligence and health technology assessment: anticipating a new level of complexity	Canada	Review	Qualitative study	Strategies: strengthen leadership, promote knowledge co-production, establish partnerships and networks, ensure organizational readiness, contextualize evidence.
Beste et al. (78)	2016	Primary care team members report greater individual benefits from long- versus short-term specialty telemedicine mentorship	United States	Original research	Quantitative study	Strategies: long-term mentorship, regular communication, collaborative learning, feedback mechanisms, integration of various guidance methods.
Mappangara et al. (79)	2020	Tele-ECG consulting and outcomes on primary care patients in a low-to-middle income population: the FIRST experience from Makassar telemedicine program	Indonesia	Original Research	Mixed methods study	Strategies: implement tele-ECG, standardize medical records, improve patient-doctor engagement.
Alenoghena et al. (80)	2023	Telemedicine: a survey of telecommunication technologies, developments, and challenges	South Africa, Niger	Review	Qualitative study	Strategies: implement new techs like blockchain, optimize resource allocation, ensure standardization.
Gizaw et al. (81)	2022	What improves access to primary healthcare services in rural communities? A systematic review	Ethiopia	Systematic review	Qualitative study	Strategies: community programs, mobile clinics, telemedicine, and funding schemes.
Yenet et al. (82)	2023	Challenges to the availability and affordability of essential medicines in African countries: a scoping review	Ethiopia	Scoping review	Mixed methods study	Strategies: strengthen governance, improve inventory control, train staff, address price issues.
Payán et al. (83)	2022	Telemedicine implementation and use in community health centers during COVID-19: clinic personnel and patient perspectives	United States	Original research	Qualitative study	Strategies: use of external resources, invest in equipment, leverage bilingual staff, provide staff training
Day et al. (84)	2021	Personalized implementation of video telehealth for rural veterans (PIVOT-R)	United States	Original research	Mixed methods study	Strategies: PIVOT-R approach, relationship building, site engagement, context assessment, goal balancing.
Nass et al. (85)	2024	Ending unequal treatment: strategies to achieve equitable health care and optimal health for All	United States	Review	Qualitative study	Strategies: community partnerships, improve healthcare workforce use health IT and enhance health literacy.
Butte et al. (86)	2024	Effectiveness of cultural sensitivity training on undergraduate students’ knowledge, self-efficacy, and ethnocultural empathy	United States	Original research	Quantitative study	/
Lee et al. (87)	2019	Specialist and family physician collaboration: Insights from primary care-based memory clinics	Canada	Original research	Quantitative study	Strategies: more time for collaboration, formalize processes, delineate roles, address barriers, use various communication methods.
Langlois et al. (88)	2016	Enhancing evidence informed policymaking in complex health systems: lessons from multi-site collaborative approaches	Mexico	Original Research	Mixed methods study	Strategies: CoPs and buddying processes, strengthening researcher-policymaker exchanges capacity building workshops.

This table summarizes key details of the 76 included publications, including authors, year, study location, study type, methods, main challenges, influencing factors, and proposed strategies for PHC capacity improvement. It provides a structured overview supporting the thematic synthesis and comparative analysis.

### Key challenges to strengthening the capacity of PHC services

#### Workforce issues

PHC workforce challenges vary significantly across development levels and between urban and rural settings (13–16). In China, a considerable proportion of community health center physicians lack formal medical education qualifications, contributing to skill gaps and limited service capability (17). Meanwhile, the continued concentration of skilled clinicians in higher-level hospitals reduces the attractiveness of PHC positions and widens the competency divide with tertiary institutions (18).

Similar disparities are observed globally. In Australia and the United States, rural communities face persistent shortages of primary care practitioners due to aging workforces and increasing healthcare demands (19–21). Thailand demonstrates one of the most acute shortages—0.8 doctors per 1,000 people, compared with an Organisation for Economic Co-operation and Development (OECD) average of 3.4 (22). Workforce pressures are further intensified by widespread burnout reported among PHC professionals in high-income countries (23–27), which negatively affects motivation and retention. These findings underscore the urgent need for targeted strategies such as rural workforce incentives, strengthened continuing professional development, and structured support to reduce burnout.

#### Financial and resource constraints

Financial and resource limitations remain a principal barrier to PHC capacity building, particularly in LMICs. Many regions continue to operate with outdated infrastructure, insufficient diagnostic equipment, and chronic staffing and supply shortages (28–30). In addition, the financial burden of accessing PHC services remains inequitable: the poorest populations experience a substantially higher risk of catastrophic health expenditure compared with the wealthiest groups (29). These constraints directly limit access to timely and high-quality PHC, exacerbating health disparities within and across countries (31, 32).

#### Policy and governance frameworks

Effective governance structures are essential to ensure accountability, quality control, and equitable PHC service provision (14, 22). Challenges related to the implementation of telemedicine and other digital tools—such as reimbursement and regulation—continue to hinder integration in several countries (8, 17, 33). Conversely, evidence from models like the Patient-Centered Medical Home (PCMH) indicates that strong governance frameworks can directly improve care quality and cost-efficiency (34). Furthermore, the experience of Indonesia demonstrates that progress toward UHC requires continuous refinement of governance systems to ensure inclusivity and fair resource allocation (35). Together, these findings highlight governance strengthening as a foundation for PHC capacity improvement.

#### Health equity and community engagement

Global inequities persist in PHC coverage: service access in low-income countries remains substantially lower (approximately 50%) than in high-income countries (approximately 90%), restricting timely and equitable access to essential care (36). Marginalized groups—including residents of geographically remote areas, refugees, and migrants—continue to face significant barriers such as transportation shortages, cultural and language constraints, and supply limitations (37–40). A WHO analysis across 47 African countries revealed only 1.55 healthcare workers per 1,000 people, far below the recommended 4.45 per 1,000 required to meet essential service needs (38). Strengthening community-based outreach models and equity-oriented service approaches remains critical to reducing these disparities.

#### Information technology

Digital health innovations hold major potential for expanding PHC capacity, particularly in rural and resource-constrained settings (41). However, persistent challenges—limited digital literacy, fragmented IT infrastructure, and interoperability concerns—continue to restrict effective uptake (42–44). For instance, despite significant growth in telemedicine use during the COVID-19 pandemic, poor connectivity and digital skills prevented many patients from benefitting fully from these services (44, 45). Privacy concerns and mistrust of artificial intelligence (AI) applications in healthcare further impede widespread application (46–48). To ensure technology enables—not replaces—human-centered care, digital health adoption must be supported by sustained investment in staff training and equitable access.

For details, please refer to Supplementary Table 1, which provides a summary of the five key challenge areas affecting PHC capacity.

### Key factors affecting the capacity of PHC services

#### Human resource

Enhancing PHC personnel capabilities requires a combination of short-term (training) and systemic (policy) strategies. Short-term training programs, such as those implemented in Malaysia, have shown success in improving providers’ ability to manage complex cases, such as elder abuse and neglect (EAN) (49). Similarly, studies in South and Southeast Asia showed that knowledge scores increased from 45 to 63% (*p* ≤ 0.001) in the intervention group, compared to a smaller increase of 6.8 to 16.1% (*p* = 0.009) in the control group (50). These results highlight the immediate effectiveness of targeted training. In addition, innovative care models, such as integrating behavioral health with chronic disease management, have been shown to enhance patient adherence and reduce emergency department visits (51, 52).

Furthermore, leadership development programs, which require longer-term investment, have demonstrated sustained improvements in team efficiency and healthcare quality across various countries (53, 54). For example, the Comprehensive Primary Care Plus (CPC+) program led to a 6% reduction in hospitalization rates, showcasing the positive impact of leadership and team-based models (55). Additionally, community-clinical linkage interventions have been linked to a 15% increase in the likelihood of reducing hemoglobin A1c (HbA1c) levels within six months, emphasizing the importance of integrated care approaches (56). However, to ensure the sustainability of these short-term gains, leadership development and integrated care models must be supported by systemic policy reforms, such as financial incentives and career development paths for PHC professionals (53, 54, 57, 58).

#### Technology and equipment upgrades

The integration of information technology (IT) and AI has enhanced the efficiency and capacity of PHC services. In LMICs, health information systems (HIS) have been shown to improve chronic disease management and patient outcomes (59). The incorporation of IT and AI into PHC services represents a combination of short-term technological upgrades and systemic policy shifts. Short-term applications, such as mobile health interventions in rural China, have demonstrated immediate reductions in systolic blood pressure among stroke patients, leading to improved short-term outcomes (60). However, broader, systemic integration of AI tools for diagnostics and treatment optimization requires comprehensive policy support to ensure equitable distribution, particularly in low-resource settings (59, 61). While AI has shown promise in enhancing clinic visit rates and medication adherence, its long-term effectiveness in reducing disease risks or mortality rates remains under evaluation (62, 63).

#### Resource integration and management

Systemic integration of resources between primary and specialized care is essential for optimizing PHC capacity. Countries such as Israel and China demonstrate that policies supporting coordinated care can significantly enhance health outcomes and reduce healthcare costs (64, 65). Integrated systems improve communication between general practitioners and specialists, reducing unnecessary treatments, care fragmentation, and hospital readmissions, while also enhancing patient outcomes (66, 67). These outcomes are particularly notable when integrated with broader policy frameworks that address issues such as healthcare financing and public-private partnerships. In contrast, short-term resource integration strategies, such as the use of telemedicine for immediate consultations, have proven effective in improving access; however, their sustainability depends on robust governance and ongoing policy adaptations (68, 69).

### Improvement strategies to increase the capacity of PHC services

#### Primary care workforce development

Effective workforce development in PHC requires both short-term training and systemic policy strategies. Short-term strategies, such as localized training programs and task-shifting, have proven particularly effective in improving healthcare delivery in rural and underserved regions. These approaches, which focus on integrating diagnostic reasoning with community health needs, allow healthcare workers to gain practical experience in their local settings (49, 70, 71). For example, task-shifting models, where general practitioners or community health workers manage prevalent diseases through brief, focused programs, have demonstrated positive results in various global health settings (18, 72).

On the other hand, systemic strategies such as providing financial incentives, career advancement opportunities, and improving working conditions are essential for retaining healthcare professionals, especially in rural areas. Evidence suggests that offering career development opportunities and community recognition can significantly improve healthcare workers’ retention rates in rural or economically disadvantaged areas (73). Collaborating with local governments to create favorable employment conditions and reduce workforce attrition is also crucial. Furthermore, leveraging low-cost e-learning tools and AI-assisted diagnostic systems provides real-time support and continuous professional development for rural healthcare providers (74, 75). These approaches are particularly beneficial in resource-constrained environments, offering a cost-effective alternative to traditional, off-site training programs.

#### Application of information technology

The application of IT and AI must be adapted to local contexts, particularly in resource-constrained settings. Short-term strategies such as AI tools that offer low-cost, low-bandwidth solutions for diagnostic support are essential for maximizing the utility of AI in remote areas. For example, in rural China, mobile health systems have been effective in managing chronic diseases, particularly in underserved populations (59). However, the full potential of AI and telemedicine can only be realized through systemic strategies that support equitable distribution. This includes funding for AI training programs, international collaborations, and subsidies aimed at ensuring vulnerable populations can access these technologies (49, 76, 77). For example, in Indonesia, the implementation of tele-electrocardiography (tele-ECG) significantly improved triage efficiency and ensured prompt referral for cardiovascular disease (CVD) patients (78). Additionally, the community telemedicine coordinator model can enhance healthcare access in rural and underserved areas by training local workers to use basic telemedicine technologies and mobile devices with low-bandwidth networks for video consultations and data sharing (79). Evidence from the Pacific Northwest, USA, indicates that telemedicine mentorship significantly improves access to specialty care and team integration (77). These short-term strategies, when coupled with systemic policies that ensure sustainable access, can greatly enhance healthcare accessibility in low-resource settings.

#### Enhancing equity in primary care services

Improving equity in PHC services requires both integrated short-term measures and long-term systemic policies (80, 81). Short-term interventions, such as telemedicine, have demonstrated immediate improvements in healthcare access in rural and marginalized populations (82). Furthermore, the establishment of community health coordinators and collaboration with local health services has proven effective in addressing specific health needs in these populations (54, 83, 84). However, to achieve long-term improvements, systemic policies are needed to strengthen resource allocation and ensure adequate healthcare facilities, medicines, and personnel in underserved regions (80, 81). Systemic interventions like health information systems (HIS) for real-time monitoring and efficient resource distribution are essential for addressing regional health disparities (59, 85).

#### Enhancing interdisciplinary collaboration

Interdisciplinary collaboration should focus on localization and resource optimization, particularly in resource-constrained settings. For instance, Canada’s Primary Care Collaborative Memory Clinics (PCCMCs) have demonstrated the impact of interdisciplinary teams in improving dementia care (86). In economically disadvantaged regions, short-term strategies, such as integrating nurses and pharmacists into primary care teams for chronic disease management, can effectively expand healthcare coverage (19). Similarly, developing locally relevant professionals, such as dietitians and mental health workers, as part of interdisciplinary teams has been shown to address specific community health needs (87). Moreover, community-centered models, such as engaging health workers as liaisons between primary and specialty care, are particularly effective in resource-limited settings. This approach enhances team collaboration, especially in chronic disease management and public health education (54, 88, 89). These strategies can be adapted globally, with context-specific collaboration models that facilitate sustainable development in PHC services.

## Discussion

### Principal findings

This scoping review synthesizes evidence from 76 global studies to delineate critical challenges and actionable strategies for strengthening PHC service competency. Key challenges include workforce shortages and maldistribution (particularly in rural and underserved regions), financial barriers and inequitable resource allocation, fragmented governance frameworks, persistent health equity gaps, and limited integration of health information technologies. To address these barriers, studies emphasized multifaceted strategies: (1) workforce capacity-building through targeted training and retention programs; (2) technology-driven innovations such as telemedicine and AI-enabled decision support systems; (3) equity-centered service models that prioritize marginalized populations; and (4) interprofessional collaboration to enhance care coordination across sectors.

### Geographic variations in challenges and transferability of findings

A deeper examination of the challenges identified in this review reveals that geographic context significantly shapes how these issues are experienced and addressed. Several challenges—such as workforce shortages, fragmented care coordination, and limited community engagement—are widely observed across both HIC and LMIC settings, making the corresponding strategies broadly transferable. For instance, continuous professional development, task-shifting, and strengthened referral systems have demonstrated effectiveness in diverse environments.

However, other challenges show substantial geographic variability, particularly those related to financing structures, governance capacity, and the availability of digital infrastructure. In HIC contexts, challenges often center around system complexity, care integration, and optimisation rather than basic resource constraints. In contrast, LMIC settings face more fundamental limitations, including inadequate facility funding, insufficient supply chains, and uneven distribution of qualified health workers. Consequently, strategies such as advanced health information technologies, data-driven decision-making, or large-scale organisational reforms require cautious adaptation when considered for resource-limited settings. Implementation in LMICs may necessitate phased approaches, simplified or low-cost digital tools, and stronger alignment with community-level capacities and sociocultural norms.

By differentiating between universally applicable findings and those requiring contextual modification, this review provides clearer guidance for policymakers and practitioners working to strengthen PHC systems across varied resource landscapes.

### Context-specific considerations and limitations of generalisability

While this scoping review synthesised global evidence on PHC capacity-building, it is important to acknowledge that a considerable proportion of the included studies originated from HICs. As a result, several identified strategies—particularly those involving advanced digital health infrastructure, specialised workforce training models, and comprehensive financing mechanisms—may not be directly transferable to LMIC contexts. The predominance of studies from HICs risks overemphasizing challenges such as technology adoption and workforce training, which may not be as pertinent in LMICs where more foundational issues like basic facility funding, supply chain deficiencies, and unequal workforce distribution prevail. Differences in resource availability, health system maturity, governance capacity, and sociocultural dynamics can significantly influence the feasibility and effectiveness of these interventions. Consequently, the generalisability of certain strategies remains limited, particularly those reliant on high-resource settings. Future research should prioritize studies from LMICs and non-English publications to ensure that capacity-building interventions are responsive to diverse health system realities.

### Strengths

This scoping review demonstrates methodological rigor through strict adherence to Arksey & O’Malley’s framework and PRISMA-ScR guidelines, with dual independent screening, blinded quality control, and iterative thematic analysis enhancing transparency and reproducibility. By synthesizing 76 studies spanning 31 countries and regions across diverse income levels, the findings offer multilevel insights into PHC systems—from digital integration in high-income settings to workforce reforms in low-resource contexts —providing policymakers with adaptable, evidence-based strategies. Additionally, explicit alignment with global agendas strengthens its relevance to advancing UHC.

## Limitations

However, limitations warrant consideration. First, reliance on English-language and peer-reviewed literature may overlook regionally critical evidence from non-English publications or LMIC governmental reports. Second, despite efforts to capture geographic diversity, the predominance of studies from HICs risks overemphasizing challenges like technology adoption while underrepresenting foundational barriers. Finally, the exclusion of gray literature may omit critical insights into national-level PHC reforms, particularly in low-resource contexts where peer-reviewed evidence is scarce. Future research should prioritize multilingual synthesis, integrate gray literature, and incorporate longitudinal assessments to address these gaps.

### Comparison of strengths and weaknesses with other studies

This review offers a more comprehensive and detailed analysis of the challenges involved in enhancing the capacities of PHC Service, providing broader coverage compared to previous studies. Many earlier studies have examined specific aspects of PHC improvement. For instance, some studies have emphasized the role of digital health tools, focusing primarily on technological innovation while overlooking broader systemic challenges such as policy reform and financial barriers (9, 43, 45, 90). Similarly, other studies have analyzed the impact of financial investment on PHC human resource development, providing valuable insights but from a somewhat narrow perspective (6, 91). Additionally, investigations into policy frameworks within specific health systems have highlighted important considerations but often failed to fully address the interconnected nature of these challenges, particularly in resource-constrained settings (69, 87, 92).

In contrast, this review synthesizes findings from 76 studies conducted in both low-resource and high-resource environments, covering multiple dimensions such as technological advancements, policy reforms, financial constraints, and workforce development. It provides a more comprehensive understanding of the complexities of PHC service, demonstrating the interconnections among these issues and offering a multidimensional framework for strengthening PHC service.

Although this review lacks the depth of quantitative analysis or empirical data provided by some meta-analyses or randomized controlled trials, its strength lies in offering a diverse and global perspective. By encompassing various geographical, economic, and policy contexts, it delivers valuable insights broadly applicable to the improvement of PHC service in different countries and regions. This contribution establishes a critical foundation for future research and policy development, emphasizing the importance of coordinated, multifaceted approaches to strengthening PHC service.

### Significance of the study

This scoping review systematically synthesizes global evidence on the multifaceted challenges impeding PHC service competency and evaluates proposed strategies to address them. By collating insights from diverse socioeconomic and geographic contexts, the study identifies transferable best practices and context-specific innovations, such as competency-based training frameworks, digital health integration, and community-participatory models. The findings aim to inform policymakers, health planners, and frontline practitioners in designing targeted interventions to enhance PHC medical service competency, optimize resource allocation, and strengthen systemic resilience. Based on the findings, specific policy recommendations include the implementation of workforce retention incentives, such as financial and professional development support for primary care providers, as well as the development of AI adoption frameworks that ensure equitable access, training, and ethical considerations in LMICs. These policy recommendations aim to address both short-term challenges and long-term system-wide improvements. Additionally, the review highlights critical gaps in evidence, including the long-term impact of technology-driven solutions and the role of policy coherence in sustaining competency-building initiatives. Ultimately, this work underscores the urgency of prioritizing PHC service competency as a catalyst for advancing global health equity and ensuring health systems are prepared to meet evolving population needs.

### Future research directions

Future research should address several limitations identified in this review to strengthen the evidence base on PHC capacity-building. First, multilingual evidence synthesis is essential to reduce language bias, as reliance on English-language sources may exclude valuable studies from regions where PHC research is published in local languages. Incorporating literature in Chinese, Spanish, Portuguese, French, and other widely used languages would provide a more comprehensive global perspective. Second, the inclusion of grey literature—such as national policy documents, program evaluations, and reports from international organisations—would help capture practical insights and implementation experiences that are often underrepresented in peer-reviewed publications. Future research should also emphasize longitudinal and comparative studies to assess the long-term impact of PHC interventions, as well as studies comparing PHC performance across diverse socio-political and economic settings. Such studies could provide critical insights into which strategies are most effective in different contexts and inform future policy design. Finally, there is a pressing need for more context-specific studies conducted in LMIC settings, where the challenges, resource constraints, and sociopolitical environments differ significantly from those in high-income countries. Such research is crucial for developing tailored, feasible, and scalable strategies to enhance PHC service capacity in diverse health system contexts.

## Conclusion

In summary, this study identifies actionable strategies to strengthen the resilience and responsiveness of PHC systems, but future research should incorporate longitudinal and comparative approaches to assess the effectiveness of these strategies over time and across varying contexts. Additionally, integrating context-sensitive and qualitative methods will deepen the evidence base, enriching our understanding of how these strategies work in different settings. By focusing on policy recommendations and locally adaptable solutions, such research will contribute to advancing equitable PHC systems that are both globally informed and responsive to local needs.

## Data Availability

The original contributions presented in the study are included in the article/Supplementary material, further inquiries can be directed to the corresponding authors.

## Glossary

PHC: Primary Health Care
IT: Information Technology
UHC: Universal Health Coverage
WHO: World Health Organization
OSF: Open Science Framework
PRISMA-ScR: Preferred Reporting Items for Systematic Reviews and Meta-Analyses Extension for Scoping Reviews
WOS: Web of Science
HICs: High-Income Countries
HRSA: Health Resources and Services Administration
OECD: Organisation for Economic Co-operation and Development
LMICs: Low- and Middle-Income Countries
CHE: Catastrophic Health Expenditure
PCMH: Patient-Centered Medical Home
LICs: Low-Income Countries
AI: Artificial Intelligence
EAN: Elder Abuse and Neglect
CPC+: Comprehensive Primary Care Plus
HbA1c: Hemoglobin A1c
HIS: Health Information Systems
ML: Machine Learning
GDP: Gross Domestic Product
tele-ECG: Tele-Electrocardiography
CVD: Cardiovascular Disease
PCCMCs: Canada’s Primary Care Collaborative Memory Clinics
