# Supplementary material for: Strengthening primary health care service competency: a scoping review of challenges, influencing factors, and enhancement strategies
Source: Front Public Health. 2026 Jan 9;13:1732011. doi: 10.3389/fpubh.2025.1732011 (PMC12829332; doi:10.3389/fpubh.2025.1732011)
Supplement: Supplementary file 1 [file Table_1.docx]

Supplementary Table 1. Summary of major challenges limiting PHC service capacity and their practical implications

| **Challenge Area** | **Key Evidence** | **Implications for PHC Capacity Improvement** |
| --- | --- | --- |
| Workforce Issues | Shortage of qualified professionals; uneven distribution;  high burnout | Workforce development, rural incentives,  professional support systems |
| Financial & Resource  Constraints | Underfunding in LMICs; outdated infrastructure;  inequitable financial burden | Increased investment, cost protection, improved  procurement |
| Policy & Governance | Regulatory and coordination gaps limit PHC reform  impacts | Strengthened oversight and policy coherence |
| Health Equity & Community  Engagement | Large disparities in coverage and access, especially for  marginalized populations | Equity-focused models, community involvement |
| Information Technology (IT) | Low digital readiness, privacy concerns, limited | Digital literacy programs, infrastructure investment |

infrastructure
