# Supplementary material for: Strengthening primary health care service competency: a scoping review of challenges, influencing factors, and enhancement strategies
Source: Front Public Health. 2026 Jan 9;13:1732011. doi: 10.3389/fpubh.2025.1732011 (PMC12829332; doi:10.3389/fpubh.2025.1732011)
Supplement: Supplementary file 3 [file Data_Sheet_2.docx]

**eAppendix 2 Search strategy**

**Timespan: 2014-01-01 to 2024-12-31 (Publication Date)**

| **Databases** | **Search string** | **Number of articles** |
| --- | --- | --- |
| **PubMed** | | |
| #1 (Step 1) | Search: ("primary health care"[Title/Abstract] OR "primary healthcare"[Title/Abstract] OR "primary care"[Title/Abstract]) OR "Community Health Service Center*"[Title/Abstract] Filters: English, Humans, from 2014/1/1 - 2024/12/31 | 81,985 |
| #2 (Step 2) | Search: ("capacit*"[Title/Abstract] OR "competenc*"[Title/Abstract] OR "capabilit*"[Title/Abstract]) AND ("building"[Title/Abstract] OR "develop*"[Title/Abstract] OR "strengthen*"[Title/Abstract] OR "increase*"[Title/Abstract]) Filters: English, Humans, from 2014/1/1 - 2024/12/31 | 156,394 |
| #3 (Step 3) | Search: (challenge*[tiab] OR barrier*[tiab] OR determinant*[tiab] OR "influencing factor*"[tiab]) AND ("Capacity Building"[Mesh] OR "Health Policy"[Mesh] OR strateg*[tiab] OR intervention*[tiab] OR telemedicine [tiab]) NOT (animal [tiab] OR mice [tiab] OR rat [tiab]) Filters: English, Humans, from 2014/1/1 - 2024/12/31 | 156,658 |
| Final search strategy | #1 AND #2 AND#3 | 751 |
| **Web of Science Timespan: 2014-01-01 to 2024-12-31 (Publication Date)** | | |
| #1 (Step 1) | TS=("primary health care" OR "primary healthcare" OR "primary care") OR TS=("Community Health Service Center*") and Preprint Citation Index (Exclude – Database) and Article or Review Article (Document Types) and English (Languages) | 132,603 |
| #2 (Step 2) | TS=(capacit* OR competenc* OR capabilit*) AND TS=(building OR develop* OR strengthen* OR increas*) OR AB=(capacit* OR competenc* OR capabilit*) AND TS=(building OR develop* OR strengthen* OR increas*) and Preprint Citation Index (Exclude – Database) and Article or Review Article (Document Types) and English (Languages) | 1,112,549 |
| #3 (Step 3) | (TS=((challenge* OR barrier* OR determinant* OR "influencing factor*") AND (strateg* OR intervention* OR "capacity building" )) NOT TS=(animal OR mice OR rat)) AND ((LA==("ENGLISH")) NOT (SILOID==("PPRN"))) and Preprint Citation Index (Exclude – Database) and Article or Review Article (Document Types) and Article or Review Article (Document Types) and English (Languages) | [515,479](https://webofscience.clarivate.cn/wos/alldb/summary/2fe1ed48-34a6-418d-bc62-2711cf2630ae-0157d43144/relevance/1) |
| Final search strategy | #1 AND #2 AND#3 | 1,616 |
| **SCOPUS** | | |
| #1 (Step 1) | ( TITLE-ABS-KEY ( "primary health care" ) OR TITLE-ABS-KEY ( "primary healthcare" ) OR TITLE-ABS-KEY ( "primary care" ) ) OR TITLE-ABS-KEY ( "Community Health Service Center*" ) AND PUBYEAR > 2013 AND PUBYEAR < 2025 AND ( LIMIT-TO ( DOCTYPE , "ar" ) OR LIMIT-TO ( DOCTYPE , "re" ) ) AND ( LIMIT-TO ( LANGUAGE , "English" ) ) | 111,174 |
| #2 (Step 2) | ( TITLE ( capacit* OR competenc* OR capabilit* ) AND TITLE-ABS-KEY ( building OR develop* OR strengthen* OR increas* ) ) OR ( ABS ( capacit* OR competenc* OR capabilit* ) AND TITLE-ABS-KEY ( building OR develop* OR strengthen* OR increas* ) ) AND PUBYEAR > 2013 AND PUBYEAR < 2025 AND ( LIMIT-TO ( LANGUAGE, "English" ) ) AND ( LIMIT-TO ( DOCTYPE, "ar" ) OR LIMIT-TO ( DOCTYPE, "re" ) ) | 954,110 |
| #3 (Step 3) | TITLE-ABS-KEY ( ( challenge* OR barrier* OR determinant* OR "influencing factor*" ) AND ( strateg* OR intervention* OR "capacity building" ) ) AND NOT TITLE-ABS-KEY ( animal OR mice OR rat ) AND ( LIMIT-TO ( DOCTYPE, "ar" ) OR LIMIT-TO ( DOCTYPE, "re" ) ) AND ( LIMIT-TO ( LANGUAGE, "English" ) ) | 623,807 |
| Final search strategy | #1 AND #2 AND#3 | 1,001 |
